# Terminal differentiation precedes functional circuit integration in the peduncle neurons in regenerating *Hydra vulgaris*

**DOI:** 10.1186/s13064-024-00194-2

**Published:** 2024-10-04

**Authors:** Alondra Escobar, Soonyoung Kim, Abby S. Primack, Guillaume Duret, Celina E. Juliano, Jacob T. Robinson

**Affiliations:** 1 Department of Bioengineering, Rice University, 6100 Main Street, Houston, TX 77005 USA; 2 Department of Electrical and Computer Engineering, Rice University, 6100 Main Street, Houston, TX 77005 USA; 3 Department of Neuroscience, Baylor College of Medicine, One Baylor Plaza, Houston, TX 77030 USA; 4 Department of Molecular and Cellular Biology, University of California, Davis, CA 95616 USA

**Keywords:** Neural regeneration, Functional neural circuit, Cell differentiation, Neurogenesis, *Hydra*, Cnidarians

## Abstract

Understanding how neural circuits are regenerated following injury is a fundamental question in neuroscience. *Hydra* is a powerful model for studying this process because it has a simple neural circuit structure, significant and reproducible regenerative abilities, and established methods for creating transgenics with cell-type-specific expression. While *Hydra* is a long-standing model for regeneration and development, little is known about how neural activity and behavior is restored following significant injury. In this study, we ask if regenerating neurons terminally differentiate prior to reforming functional neural circuits, or if neural circuits regenerate first and then guide the constituent naive cells toward their terminal fate. To address this question, we developed a dual-expression transgenic *Hydra* line that expresses a cell-type-specific red fluorescent protein (tdTomato) in ec5 peduncle neurons, and a calcium indicator (GCaMP7s) in all neurons. With this transgenic line, we can simultaneously record neural activity and track the reappearance of the terminally-differentiated ec5 neurons. Using SCAPE (Swept Confocally Aligned Planar Excitation) microscopy, we monitored both calcium activity and expression of tdTomato-positive neurons in 3D with single-cell resolution during regeneration of *Hydra’s* aboral end. The synchronized neural activity associated with a regenerated neural circuit was observed approximately 4 to 8 hours after expression of tdTomato in ec5 neurons. These data suggest that regenerating ec5 neurons undergo terminal differentiation prior to re-establishing their functional role in the nervous system. The combination of dynamic imaging of neural activity and gene expression during regeneration make *Hydra* a powerful model system for understanding the key molecular and functional processes involved in neural regeneration following injury.

**Supplementary Information:**

The online version contains supplementary material available at 10.1186/s13064-024-00194-2.

## Background

Neural regeneration capacity widely varies across animal species. The regeneration of nervous systems range from the growth of innervated limbs in salamanders [1, 2] or recovery from spinal cord injuries [3, 4] in certain vertebrates to full nervous system regeneration in *Hydra* [5–7] or flatworms [8, 9]. Mammals, on the other hand, exhibit limited regenerative abilities, along with a complex immune response that slows neural regrowth [10]. Understanding how newly-generated neurons rebuild functional neural circuits can help the development of neural repair therapies for humans. The cnidarian polyp *Hydra* has a simple neural structure, extensive neuronal regenerative capabilities [7, 11, 12], established genetic tools [13, 14], and is an emerging model organism for neuroscience [15, 16]. These features make *Hydr*a an excellent model for interrogating the cellular dynamics of functional regeneration of neural circuits.

*Hydra* has a simple radial body plan organized around a single oral-aboral axis of symmetry, with the hypostome and tentacles at the oral end and the peduncle and basal disk at the aboral end. The *Hydra* body has two epithelial monolayers, the inner endoderm and outer ectoderm, separated by an extracellular matrix [17]. *Hydra* has two separate nerve nets, one embedded in each of the epithelial layers. Neurons run along the entire length of the body, with a higher neuron density in the hypostome (oral end) and the peduncle and basal disk (aboral end) [12]. Critically, *Hydra* has a defined repertoire of behaviors associated with four non-overlapping neural circuits: rhythmic potential 1 (RP1), rhythmic potential 2 (RP2), contraction burst (CB), and the sub-tentacular network (STN) [18]. In this study we focus on the recovery of the CB circuit during regeneration; this circuit is associated with longitudinal contractions, a behavior that is frequent and quantifiable. The CB circuit is composed of neurons that run the length of the ectodermal epithelium, including the ec5 neurons found in the peduncle [18, 19]. This particular group of neurons are relatively easy to track via time lapse imaging due to the size and the synchronous group activity [20].

The transcriptional state of *Hydra* neuron subtypes has been profiled using single cell RNA-sequencing [19, 21]. Similar to findings in *C. elegans* [22], the *Hydra* neurons are best defined by unique combinatorial expression of specific genes, including transcription factors and neuropeptides [21, 23, 24]. The neuronal subtypes participating in the CB circuit are defined by combinatorial expression of various paralogs of the Hym176 neuropeptide gene (Hym176A-E) [24–26]. Since ec5 neurons specifically express the neuropeptide Hym176C, this gene's regulatory region was used to drive ec5-specific expression [19, 26].

*Hydra* is a long-standing regeneration model and is an emerging model for neuroscience. However, little is known about the restoration of neural activity and behavior following significant injuries. During the neural circuit regeneration process, two key events occur: terminal cell differentiation and synchronization of cell activity [19, 21, 27–30]. However, the order of the two events is unknown. In this study, we ask whether regenerated neurons reach a terminal cell fate and then functional neural circuits are restored or if an initial regeneration of neural circuits guides the differentiation of the constituent naive cells.

Here, we used the regulatory region of *Hym176C* (G016165) to drive the ec5-specific nuclear expression of tdTomato and the regulatory region of *tba1c* (G019559) [21] to drive the expression of GCaMP7s [31] in all neurons. This allowed us to perform cell-type specific 3D imaging of neural activity in both uninjured and regenerating *Hydra.* We first confirmed that ec5 neurons are part of the CB circuit, and then observed the regeneration of the CB circuit by tracking the neural activity and reappearance of ec5 neurons after amputation of the aboral end. We found that ec5 neurons terminally differentiate before they synchronize with neighboring neurons. This suggests that positional rather than neural activity cues play the dominant role in guiding neuronal cell fate in this circuit. This study provides the foundational tools and conceptual framework to better understand the molecular mechanisms that underlie the regeneration of functional neural circuits.

## Methods

### Generation of *Tg(Hym176c:tdTomato,tba1c:GCAMP7s)*^cj1-in^ transgenic strain

**Fig. 1:**
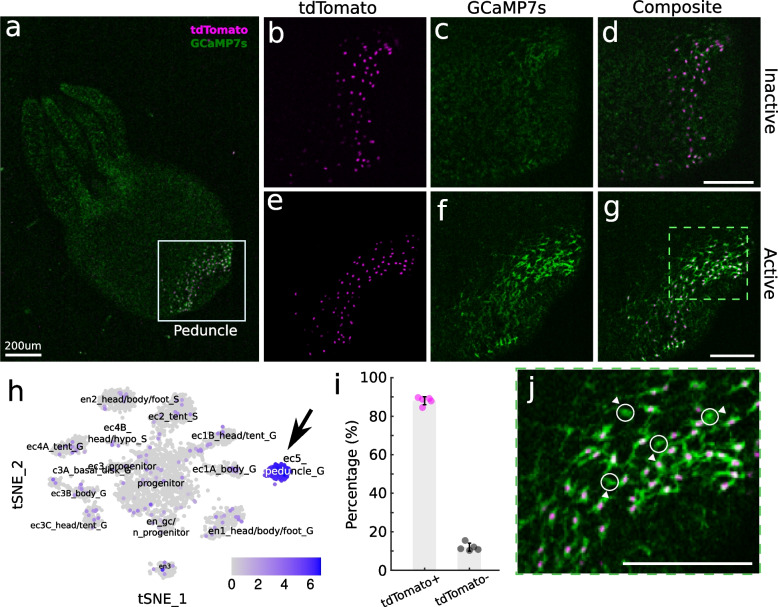
ec5 neurons are part of the Contraction Burst (CB) neural circuit (**a**) Fluorescence image of a *Tg(hym176c:tdTomato,tba1c:GCAMP7s)*^cj1-in^ *Hydra* expressing nuclear-localized tdTomato in a subpopulation of neurons in the foot and GCaMP7s in all the neurons. **b**-**g** High magnification images of *Hydra*’s foot (white box in panel a) showing dual expression of (**b**) tdTomato-positive ec5 neurons in magenta and **c** GCaMP7s in all neurons during inactive state (**d** - Composite image). When active, the ec5 neuron nuclear tdTomato expression is unchanged (**e**) while GCaMP fluorescence increases (**f**). **h** t-SNE representation of single neuron transcriptomes collected from *Hydra* (public data available from Siebert et al. [19]). The arrow indicates the ec5 neurons highly expressing *Hym176C*. The numbers on the colorbar correspond to log-normalized feature counts. **i** The percentage of tdTomato-positive (magenta, mean = 88.02, SD = 2.08) and tdTomato-negative (black, mean = 11.98, SD = 2.08) neurons that express GCaMP7s in the peduncle. Error bars show standard deviation. **j** High magnification of (**g**). Circles with arrowheads indicate tdTomato-negative neurons in the peduncle. Scale bar for all panels: 200 µm

**Fig. 2:**
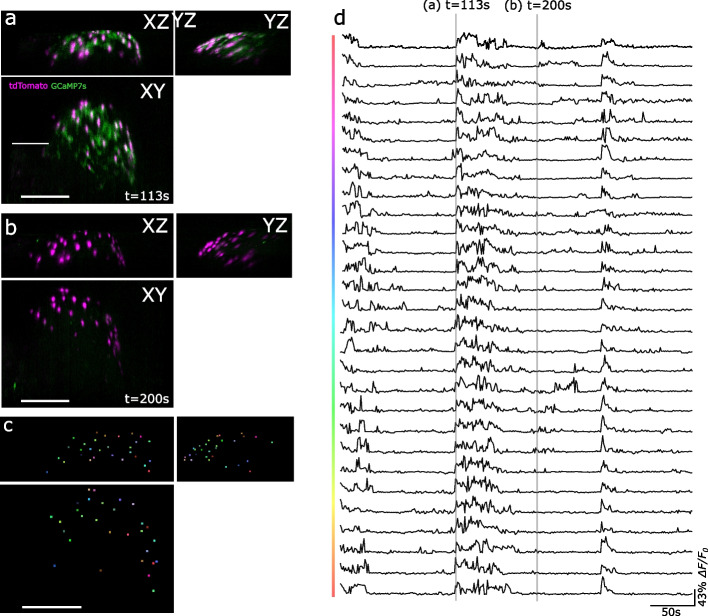
SCAPE 2.0 microscopy enables volumetric tracking of cell type specific neural activity. **a**-**b** Maximum intensity projections along x, y, and z axes acquired from volumetric SCAPE imaging of the peduncle of a behaving *Hydra*. Each panel shows a select time during this representative imaging session. Green shows calcium activity (GCaMP7s). Magenta shows the nuclei of ec5 cells (nuclear-localized tdTomato). Scale bar = 200 µm. **c** Colored dots indicate individual neurons that are tracked over the course of recording. Scale bar = 200 µm. **d** Time course of the GCaMP7s fluorescence measured in each of the labeled neurons in panels **a** and **b**. The shaded regions in gray correspond to the time points used to generate the images in panels **a** and **b**

*Hydra* transgenic line *Tg(Hym176c:tdTomato,tba1c:GCAMP7s)*^cj1-in^ was created by microinjection of embryos using an established protocol [13, 32]. A single plasmid containing two expression cassettes was built for embryo microinjection: 1) *nls-tdtomato* driven by a 2022 bp section of the *Hym176C* (G016165) regulatory region, which drives specific expression in ec5 neurons from the peduncle (Fig. 1h), and 2), *gcamp7s* driven by a 1901 bp section of the *tba1c* (G019559) regulatory region for pan-neuronal expression [21]. The plasmid solution was injected into *Hydra vulgaris* AEP 1-cell stage embryos using an Eppendorf FemtoJet 4x and Eppendorf InjectMan NI 2 microinjector (Eppendorf; Hamburg, Germany) under a Leica M165 C stereo microscope (Leica Microscopes, Inc; Buffalo Grove, Il).

Hatchlings were screened to select tdTomato-positive polyps. Continuous asexual reproduction cycles of hatchlings with mosaic transgenic tissue yielded transgenic animals with nearly uniform transgenic expression.

### *Hydra* strain maintenance

All animals were maintained using standard procedures adapted from the Steele Lab (UC Irvine). All experiments were performed using the transgenic *Hydra* line *Tg(Hym176c:tdTomato,tba1c:GCAMP7s)*^cj1-in^. *Hydra* polyps were cultured at standard conditions and incubated at 18°C with *Hydra* media under 12h:12h light:dark light cycles. *Hydra* media was made with 1000X dilution of 1.0M CaCl_2_, 0.1M MgCl_2_, 0.03M KNO_3_, 0.5M NaHCO_3_, 0.08M MgSO_4_. Polyps were fed three times per week with freshly hatched *Artemia nauplii* (Brine Shrimp Direct) and cleaned 6 hours post feeding with fresh *Hydra* media. The animals were starved 24 hours prior to surgical resections.

### Animal resections

*Hydra* were placed in a petri dish filled with *Hydra* media prior to amputation. Animal resections were performed using a scalpel (X-ACTO blade #10) and bisections were made perpendicular to the oral-aboral axis and halfway between the oral and aboral ends. The aboral end was discarded and the oral end was used for foot regeneration experiments. Regenerating *Hydra* were kept at 18°C for four hours to allow wound closure before imaging.

### Imaging configuration

Fluorescence imaging was performed by placing a single resected *Hydra* between two glass coverslips (University Wafer Fused Silica Catalog No. 1013, EVER Scientific Catalog No. 112460) separated by a 100 µm spacer. Dual-color volumetric imaging was performed in 20-minute sessions at 4 or 8 hour intervals for 44 hours post amputation (hpa) at 100 fps (1.3 VPS) using Swept Confocally Aligned Planar Excitation (SCAPE) 2.0 microscopy. The system was built following the design and configuration from Voleti *et al.* 2019 with assistance and support from Elizabeth Hillman’s laboratory at Columbia University [33]. The configuration consisted of an 20X Olympus (XLUMPLFLN 20XW 20x/1.00NA) as the primary objective lens, (for specimen illumination and light collection), followed by a Nikon 20x/0.75NA and Nikon 10x/0.45NA as the second and third objective lenses according to the SCAPE system nomenclature. The system used in all experiments had an effective detection NA of 0.23 and used Andor Zyla 4.2+ as the detector for imaging sessions. The microscope system provided oblique light sheet illumination across the field of view (800 µm x 350 µm x 100 µm). Oblique illumination was achieved by enabling the light sheet to enter the back aperture of the primary objective lens with an offset of 7 mm from the center of the objective. Coherent Obis LX 488 nm and 561 nm lasers were used as excitation laser sources for green (GCaMP7s) and red (tdTomato) channels respectively at an output power of 5 mW/mm^2^. Excitation and emission filters used for all dual color imaging experiments are listed in Supplementary Table 1. Epifluorescence microscopy was used for whole-animal imaging on a confocal microscope (Nikon A1) (Fig. 1).

### Image processing and cell tracking

For injured *Hydra*, both channels were acquired and registered to one another and exported to 16 bit tiff format using a custom MATLAB GUI provided by Elizabeth Hillman’s laboratory at Columbia University [33]. Imaris software and 3D Viewer plugin from Fiji [34] were used to visualize the channel-merged image sequence in 3D. Then, maximum intensity projections from recordings were imported to Fiji and contrast was adjusted. Particle-tracking algorithm TrackMate v6.0.2 and ManualTracking plugins [35] from Fiji were used to track single cell activity. Prior to tdTomato expression, GCaMP7s signals were manually tracked using frame to frame analysis (ManualTracking) on Fiji. Posterior to tdTomato expression, GCaMP7s fluorescence traces corresponding to tdTomato-negative neurons continued to be manually annotated using ManualTracking while GCaMP7s traces from tdTomato-positive neurons were automatically tracked using TrackMatev6.0.2 (LoG detector sigma:15-20; Simple LAP tracker) followed by manual corrections.

The same imaging procedure was used for uninjured *Hydra*. Autoregressive motion with MaxGapSize=3 was used for particle tracking. Cells that fall outside the FOV for more than 50% of the recorded time were not included in the calcium traces shown in Fig. 2.

### Statistical analysis of neural activity

**Fig. 3:**
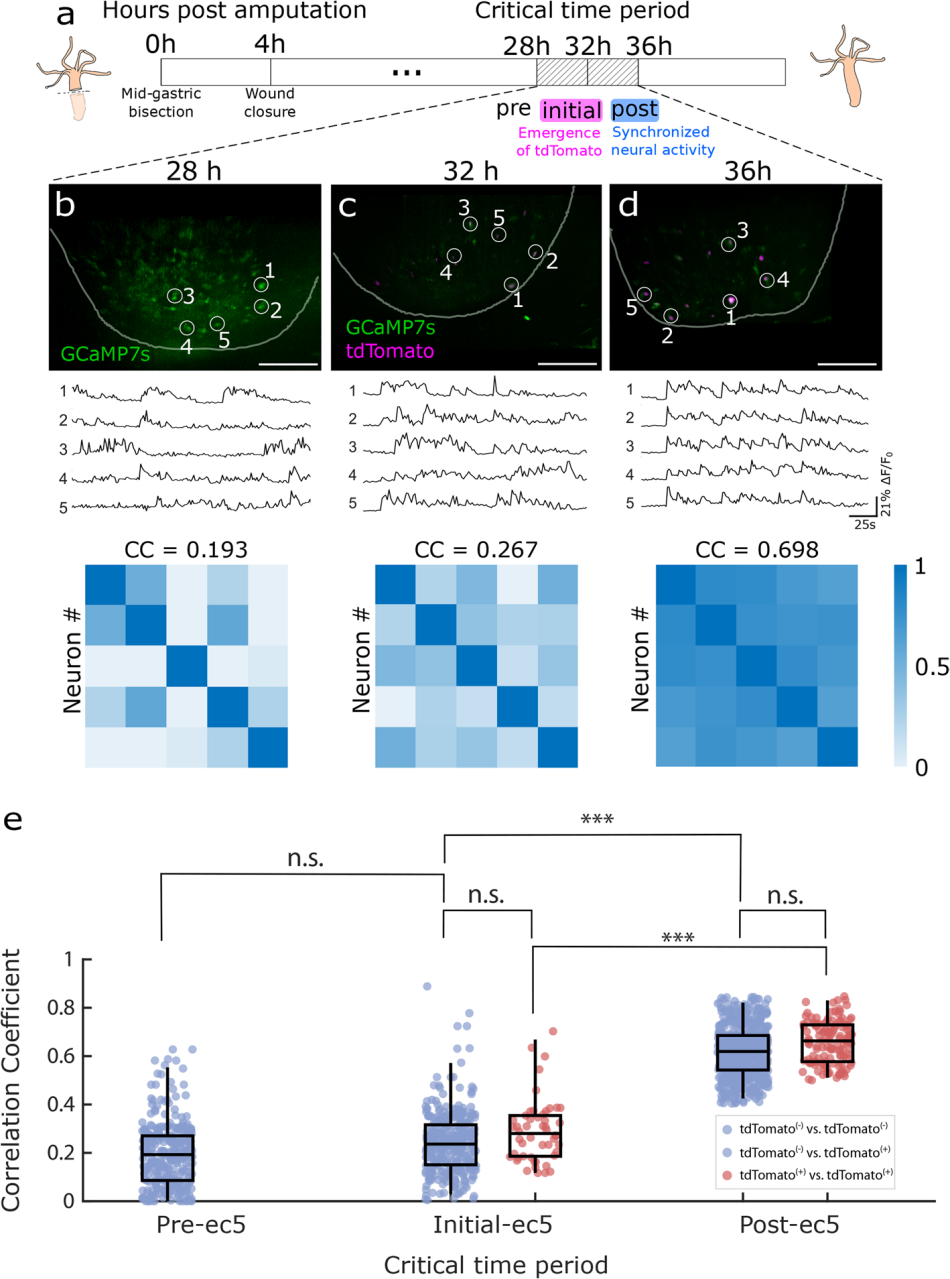
Ec5 neuron differentiation precedes synchronization in neural activity during peduncle regeneration. **a** Schematic representation of foot regeneration timeline after a mid-gastric bisection. **b**-**d** (Top row) Representative composite fluorescence image of nuclear tdTomato (magenta) and GCaMP7s (green) from the same *Hydra* at indicated time points, scale bar = 100 μm. (Middle row) GCaMP7s traces extracted from the circled neurons at time points corresponding to the top row. Numbered circles indicate the neurons of which spontaneous GCaMP7s activities are plotted. (Bottom row) Cross-correlation matrix of the circled neurons as an indicator for synchrony at time points corresponding to the top and middle row with average correlation coefficient (CC). **e** Dots represent correlation levels between two arbitrary neurons’ activity. Correlation between two tdTomato-positive neurons is labeled pink. Correlation between two tdTomato-negative neurons, or between one tdTomato-positive and one tdTomato-negative neuron is labeled blue. Top line of the box corresponds to the third quantile (75%), the middle line of the box corresponds to the median (50%), and the bottom line of the box corresponds to the first quantile (25%). The higher end of the whisker corresponds to 98%, and the lower end of the whisker corresponds to 2%. (*n* = 5 *Hydra,* n.s. = not significant, *** *p* ≤ 0.001, Kruskal-Wallis with Dunn-Sidak post hoc test (MATLAB))

For all animals (*n* = 5), single cell calcium activity traces from all regeneration time points (8-44 hpa, Supplemental Figure 1) were analyzed using MATLAB (Fig. 3). Relative GCaMP7s intensity was normalized using the minimum and maximum pixel intensity. To evaluate the level of synchrony in the neurons of interest, all obtained traces were compared to each other by measuring the linear dependence between two arbitrary cells’ activity and assigning a Pearson correlation coefficient (calculated with the MATLAB function *corrcoef*). The linear relationship between two arbitrary time series was performed on a 15 min time series, using a scale of 0 to 1 with 1 indicating a higher correlation. The correlation coefficients were then used to build a correlation matrix for every regeneration time point. To compare the difference of correlation coefficients within and between the regeneration time points (Fig. 3e), we performed a Kruskal-Wallis test with post hoc Dunn-Sidak test.

## Results

### Ec5 neurons are a part of the Contraction Burst (CB) circuit

In order to first test if ec5 neurons are a part of the CB neural circuit, we created the transgenic line, *Tg(Hym176C:tdTomato,tba1c:GCAMP7s)*^cj1-in^. TdTomato is driven by the regulatory region of *Hym176C*, which drives specific expression in ec5 neurons, and GCaMP7s is driven by the *tba1c* regulatory region for pan-neuronal expression (See Methods and Materials for details). This allowed us to simultaneously monitor the identity and activity of the ec5 neurons via tdTomato expression and GCaMP7s expression, respectively (Fig. 1).

We confirmed that only ectodermal neurons in the peduncle expressed tdTomato and that GCaMP7s successfully reported calcium activity by measuring the fluorescence levels during contractions. Similar to prior calcium imaging [20, 36], the neuronal activity in the peduncle showed increased activity during contractions (Fig. 1c,f, Supplemental Video 1). We also found that the tdTomato-positive (tdTomato^+^) neurons were a subpopulation of the neurons that were coactive during contractions, providing further evidence that ec5 neurons are in fact a part of the CB circuit (Fig. 1b-g). These tdTomato^+^ ec5 neurons represent 88% of the transgenic neurons found in the peduncle that showed increased calcium activity during contractions (*n* = 5 *Hydra*, Fig. 1i). The remaining tdTomato-negative (tdTomato^-^) peduncle neurons (Fig. 1i,j) in the CB circuit are likely the ec1A neurons that extend from the body column [19, 24].

## Volumetric fluorescent imaging establishes basal levels of synchronization among neurons in the CB circuit

Our next goal was to use the *Tg(Hym176C:tdTomato,tba1c:GCAMP7s)*^cj1-in^ transgenic line to track ec5 neuron activity during regeneration to identify when normal circuit activity resumes. To determine when the CB circuit has fully regenerated, we first established the degree of synchrony of a functional CB circuit in an uninjured animal. We imaged the spontaneous calcium activity of the tdTomato^+^ neurons using SCAPE 2.0 microscopy [33]. This dual-color fast light sheet imaging technique achieves a volumetric frame rate of 1.3 volumes per second (VPS), which is sufficient to accurately capture the calcium dynamics of ec5 neurons in 3D during contractions (Fig. 2a,b). Using IMARIS and Fiji, we then tracked individual ec5 neurons to extract the respective calcium activity (See Methods, Fig. 2c-d, Supplemental Video 1-3). The cross-correlation coefficient (CC) was calculated from the obtained calcium traces to measure the level of synchrony in the neural activity (Fig. 2d). The CC value between the ec5 neurons (*n* = 29 neurons in one *Hydra*) in an uninjured *Hydra* was 0.84 +/- 0.07 (mean +/- SEM). CC values below that threshold would indicate that the CB circuit has not fully recovered from injuries.

## Ec5 neuron differentiation precedes neural synchronization during regeneration

After measuring synchrony in an uninjured CB circuit, we investigated the temporal relationship between synchronous neural activity and terminal cell-fate marker expression as the CB circuit regenerates. Two scenarios were envisaged: (1) newly-regenerating ec5 neurons are functionally integrated into circuits and synchronize before completing terminal differentiation, or (2) ec5 neurons express differentiation markers before functional integration into the CB circuit. Since *Hym176C* is a marker of differentiated ec5 neurons, tdTomato expression under the *hym176C* regulatory region indicates a completed differentiation.

Since ec5 neurons are located in the peduncle [19, 26], we were able to produce an ec5-free *Hydra* by bisecting the animal perpendicular to the oral-aboral axis at the midpoint between the head and foot, and discarding the lower part of the animal. In approximately 48 hours after this injury, the foot fully regenerates, with new neurons produced from the interstitial stem cells that reside among the ectodermal epithelial cells in the body column. Over the course of regeneration, we tracked the reappearance of ec5 neurons (tdTomato expression) and the activity of the CB circuit (GCaMP7s fluorescence) in the peduncle. Every 4 or 8 hours post-amputation (hpa), 20-minutes fluorescent activity recordings were acquired to monitor circuit reformation (*n* = 5 *Hydra*).

In a representative experiment shown in Fig. 3a-d, we randomly selected 5 neurons from one regenerating *Hydra* to evaluate the level of synchrony. For this particular animal, we observed unsynchronized neural activity with no tdTomato-positive neurons (Fig. 3b, Supplemental Figure 1, Supplemental Video 4) from 0 - 28 hpa. At 32 hpa, tdTomato-positive neurons began to appear, but these neurons exhibited a low level of synchrony (Fig. 3c, Supplemental Video 5). 4 hours later at 36 hpa, we observed an increase in the number of tdTomato-positive neurons along with a large increase in synchronization (Fig. 3d, Supplemental Video 6). We performed this experimental approach in five different animals, up to 44 hpa (Supplemental Figure 1). Due to the variation in the timing of TdTomato expression across multiple animals and sampling intervals, we defined “initial-ec5” as the time point when tdTomato-positive neurons were first observed at a recording session (*n* = 5 *Hydra*). With this alignment, we found a critical window of 4 to 8 hours that separates the first detection of tdTomato-positive cells and the synchronization of the CB circuit, which we called the “critical time period” (Fig. 3a). Critical time period consists of 3 time points: 1) pre-ec5 (4 hours prior to tdTomato expression), 2) initial-ec5 (start of tdTomato expression) and 3) post-ec5 (4 to 8 hours after the first observed tdTomato expression). At the pre-ec5 time point, the activity of the neurons in the regenerating foot were on average completely unsynchronized (Fig. 3e, n = 5 *Hydra*, 56 neurons, CC = 0.194+/-0.07 (mean +/- SEM)). At the initial-ec5 time point, the synchrony of the neurons was low (*n* = 5 *Hydra*, 63 neurons, CC = 0.249+/-0.07) even with the presence of tdTomato-positive neurons. The level of synchrony increased significantly (*n* = 5 *Hydra*, 86 neurons, CC = 0.625+/-0.04) at the post-ec5 time point along with an increase in the number of tdTomato-positive neurons. However, they were less synchronized compared to the neurons in uninjured animals (CC = 0.84 +/- 0.07 (mean +/- SEM)). Together, these data demonstrate that ec5 neurons fully differentiate before functional integration into the CB circuit.

## Conclusion and discussion

Although specification of neurons and assembly of neural circuits during development is relatively well studied, these processes are not as well understood during regeneration after injury. *Hydra* has a unique combination of advantages compared to existing neuronal regeneration models, notably that we can image the activity of the entire nervous system with single-cell resolution. In this study, we built new tools and leveraged volumetric imaging to examine the temporal relationship between neuronal differentiation and the recovery of functional neural circuit activity in a regenerating *Hydra*. We found that the ec5 neurons reach their terminal cell fate before they functionally integrate into the CB circuit. These data suggest that during regeneration of the aboral end, cues from surrounding cells direct differentiation of stem cells into the appropriate ec5 neurons rather than existing circuit activity directing this fate. Expanding these experiments to all other neuronal subtypes in the future will provide us insight into whether this regeneration process is generalizable to the entire *Hydra* nervous system. Further work should be done to identify injury-induced differentiation signaling.

One possible reason that terminal neuronal differentiation precedes circuit-level functional recovery could be due to the expression of innexin2. Innexin2 is a protein known to form gap junctions that coordinate contractions in the *Hydra* peduncle [25], where ec5 neurons are found. Given that the ec5 neurons fire simultaneously, most likely via gap junctions, the unsynchronized neural activity suggests the involvement of innexin2 in regenerating neurons. In order to support this claim, we analyzed the expression dynamics of *Innexin2* and *Hym176C* during ec5 differentiation using the recently-published single cell data (Supplemental Figure 2). We found that both *Innexin2* and *Hym176C* are expressed during terminal differentiation. However, it may be difficult to resolve the exact timing of gene expression with a temporal resolution of a few hours, which is the time scale over which we observe differences in cell differentiation and functional recovery. On a related note, more work can be done to understand the relationship between the neuronal network size and the level of synchronization. A previous study on neural activity of *Hydra* peduncle neurons upon thermal stimulation shows the peduncle network functions normally even with only half the number of neurons of the original network size [20]. However, it is currently unclear whether there is a minimum threshold of the number of neurons required to achieve synchronization.

Future work should focus also on comparative and evolutionary studies. Other animals, ranging from cnidarians to amphibians, have high nervous system regenerative capabilities. Prior reports across species suggest that functional recovery most likely occurs after cellular regeneration. However, as we understand it, no previous study had definitively addressed this question [37–39]. Thus, to the best of our knowledge, this work represents the first study clearly demonstrating the relationship between the regeneration of a specific neural cell type and the recovery of neural circuit activity. However, even though it is highly possible that other regenerating species and *Hydra* alike share the regeneration mechanism that functional circuit recovery happens after neurons mature [40], it is not yet clear if this is a generalizable mechanism.

The transgenic *Hydra* strain *Tg(Hym176C:tdTomato,tba1c:GCAMP7s)*^cj1-in^ created here is the first to allow for simultaneous measurement of neural activity and cell type-specific gene expression during active regeneration. Combining this dual-reporter system with high-speed volumetric imaging brings *Hydra* into the small but growing group of neuroscience research organisms [41–43] where we can perform functional volumetric imaging with cell-type specificity. Coupled with *Hydra’s* unique and extensive regenerative capabilities, this can unlock opportunities to probe circuit reformation dynamics between multiple cell types during regeneration. One future direction is to investigate the temporal relationship between regenerating multiple neuronal subsets that most likely constitute a single CB circuit, namely ec1A, ec1B, and ec5, that are considered to regulate contractions in *Hydra* [26]. Harnessing the recent single-Cell RNA-Seq and ATAC-Seq data [44], we can identify other strong reporters of neuronal fate for different neural circuits. The approach proposed here can validate their role and establish the temporal parameters to build a full-organismal chronological expression profile during regeneration. Overall, the ability to monitor neural activity with cell type specificity combined with *Hydra*’s unique regenerative capabilities raises *Hydra* as a model organism for studying comprehensive neural circuit regeneration in a simple yet highly dynamic nervous system.

## Supplementary Information

Supplementary Material 1: Supplemental Table 1. Optic filters used in regeneration experiments.

Supplementary Material 2: Supplemental Figure 1. Cell count and synchrony in neural activity for n = 5 *Hydra*. (Left column) The number of tdTomato-positive neurons in magenta, and the average correlation coefficient (CC) tracked over the course of 8 - 44 hpa. The blue shaded region indicates the critical time period. (Middle column) The number of tdTomato-positive neurons in magenta and the CC values in blue with average shown in inverted triangle during the critical time period. (Right panel) The number of tracked cells during the critical time period.

Supplementary Material 3: Supplemental Figure 2. Dynamics of *Innexin2, Myb, *and*Hym176C* in pseudotime for differentiation (public data from [21]). Left to right on the x axis corresponds to stages in differentiation. Y axis corresponds to log-normalized feature count scaled from 0 to 1 for comparison.

Supplementary Material 4: Supplemental Figure 3. Correlation coefficient comparison between time points and between cell groups. Note that in the pre-ec5 time point, there are only tdTomato-negative neurons. Top line of the box corresponds to the third quantile (75%), middle line of the box corresponds to the median (50%), and the bottom line of the box corresponds to the first quantile (25%). The higher end of the whisker corresponds to 98%, and the lower end of the whisker corresponds to 2%. Kruskal-Wallis test with post hoc Dunn-Sidak test was used to evaluate statistical significance.

Supplementary Material 5: Supplemental Video 1. Single cell activity of an uninjured *Hydra’s *foot. Playback speed 1X.

Supplementary Material 6: Supplemental Video 2. Single cell tracking of an uninjured *Hydra’s *foot. Playback speed 1X.

Supplementary Material 7: Supplemental Video 3. Single cell activity traces of an uninjured *Hydra’s *foot. Playback speed 1X. 2 cells that were outside the FOV for more than >67% of the recorded time were not included in the tracking process.

Supplementary Material 8: Supplemental Video 4. Single cell activity of an injured *Hydra*at the critical time point “t=-4hr” showing unsynchronized activity of GCaMP7s-positive cells and absence of ec5 neurons. Playback speed 1X.

Supplementary Material 9: Supplemental Video 5. Single cell activity of a regenerating *Hydra* at the critical time point “t=0hr” showing unsynchronized activity of GCaMP7s-positive cells and emergence of ec5 neurons. Playback speed 1X.

Supplementary Material 10: Supplemental Video 6. Single cell activity of a regenerating *Hydra* at the critical time point“t=+4hr” showing synchronized activity of GCaMP7s-positive cells and ec5 neurons. Playback speed 1X.

## Data Availability

No datasets were generated or analysed during the current study.
