# Supplementary material for: Terminal differentiation precedes functional circuit integration in the peduncle neurons in regenerating Hydra vulgaris
Source: Neural Dev. 2024 Oct 4;19:18. doi: 10.1186/s13064-024-00194-2 (PMC11452936; doi:10.1186/s13064-024-00194-2)
Supplement: Supplementary file 1 — Supplementary Material 1: Supplemental Table 1. Optic filters used in regeneration experiments. [file 13064_2024_194_MOESM1_ESM.pdf]

Supplemental Table 1. Optic filters used in regeneration experiments.

Filter type	Description	Vendor
Excitation/emission dichroic	500 nm long pass	FF495-Di03-25x36 (Semrock)
Emission	495 nm long-pass dichroic	FF01-496/LP-25 (Semrock)
Image splitter dichroic	560 nm long-pass dichroic	FF560-FDi01-25x36 (Semrock)
Green emission filter	525 +/- 50 nm bandpass	FF01-525/50-25 (Semrock)
Red emission filter	618 +/- 50 nm bandpass	FF01-618/50-25 (Semrock)
Laser combining dichroic	556 nm short-pass dichroic	FF556-SDi01-25x36 (Semrock)
Dichroic Beam Splitter	488/561 nm dichroic beamsplitter	Di01-R488/561-25x36 (Semrock)
