# Supplementary figures and images for: Terminal differentiation precedes functional circuit integration in the peduncle neurons in regenerating Hydra vulgaris
Source: Neural Dev. 2024 Oct 4;19:18. doi: 10.1186/s13064-024-00194-2 (PMC11452936; doi:10.1186/s13064-024-00194-2)

# Hydra 1

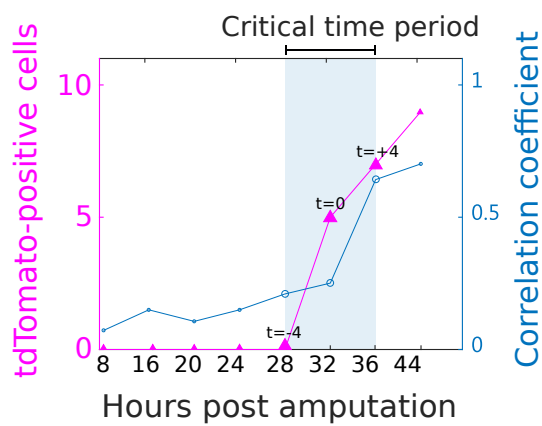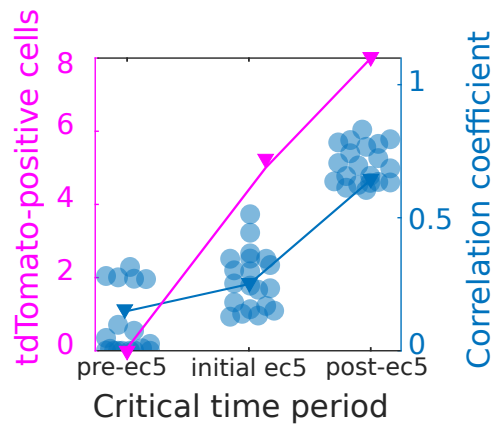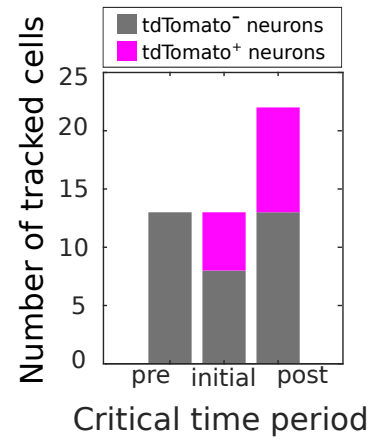

# Hydra 2

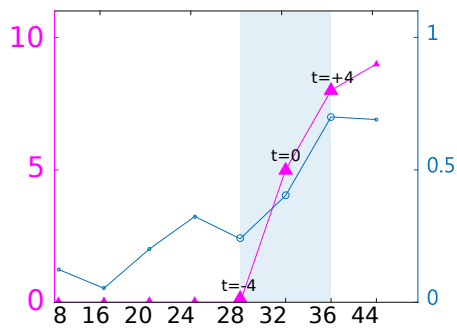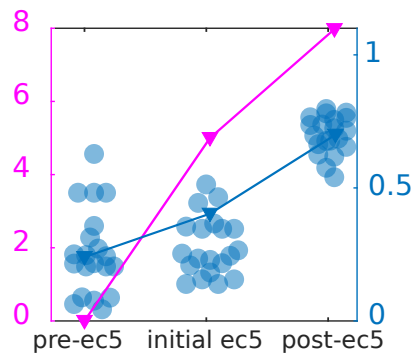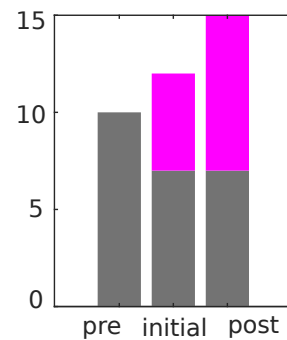

# Hydra 3

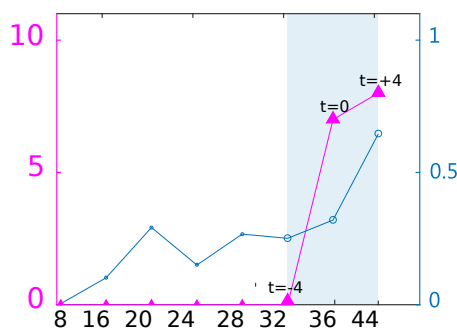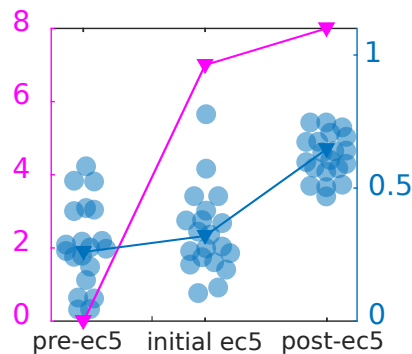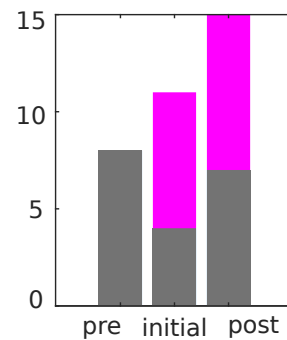

# Hydra 4

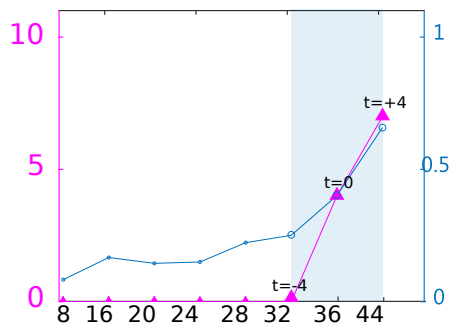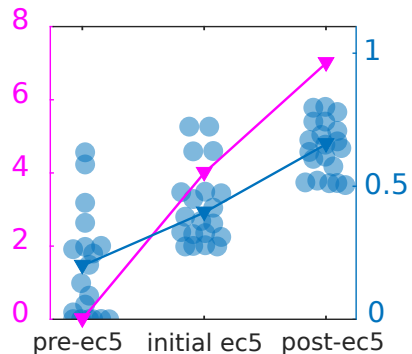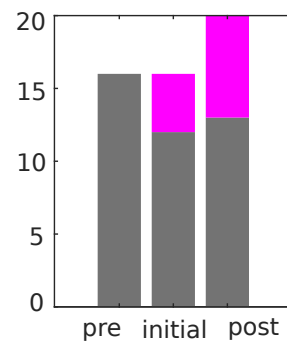

# Hydra 5

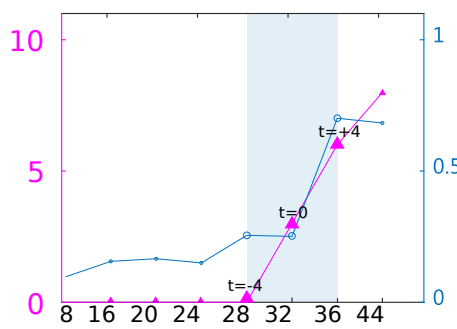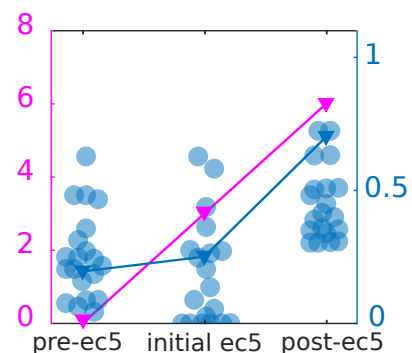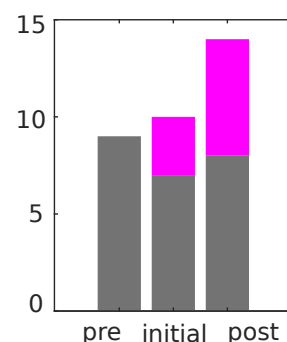

Supplement: Supplementary file 2 — Supplementary Material 2: Supplemental Figure 1. Cell count and synchrony in neural activity for n = 5 Hydra. (Left column) The number of tdTomato-positive neurons in magenta, and the average correlation coefficient (CC) tracked over the course of 8 - 44 hpa. The blue shaded region indicates the critical time period. (Middle column) The number of tdTomato-positive neurons in magenta and the CC values in blue with average shown in inverted triangle during the critical time period. (Right panel) The number of tracked cells during the critical time period. [file 13064_2024_194_MOESM2_ESM.pdf]

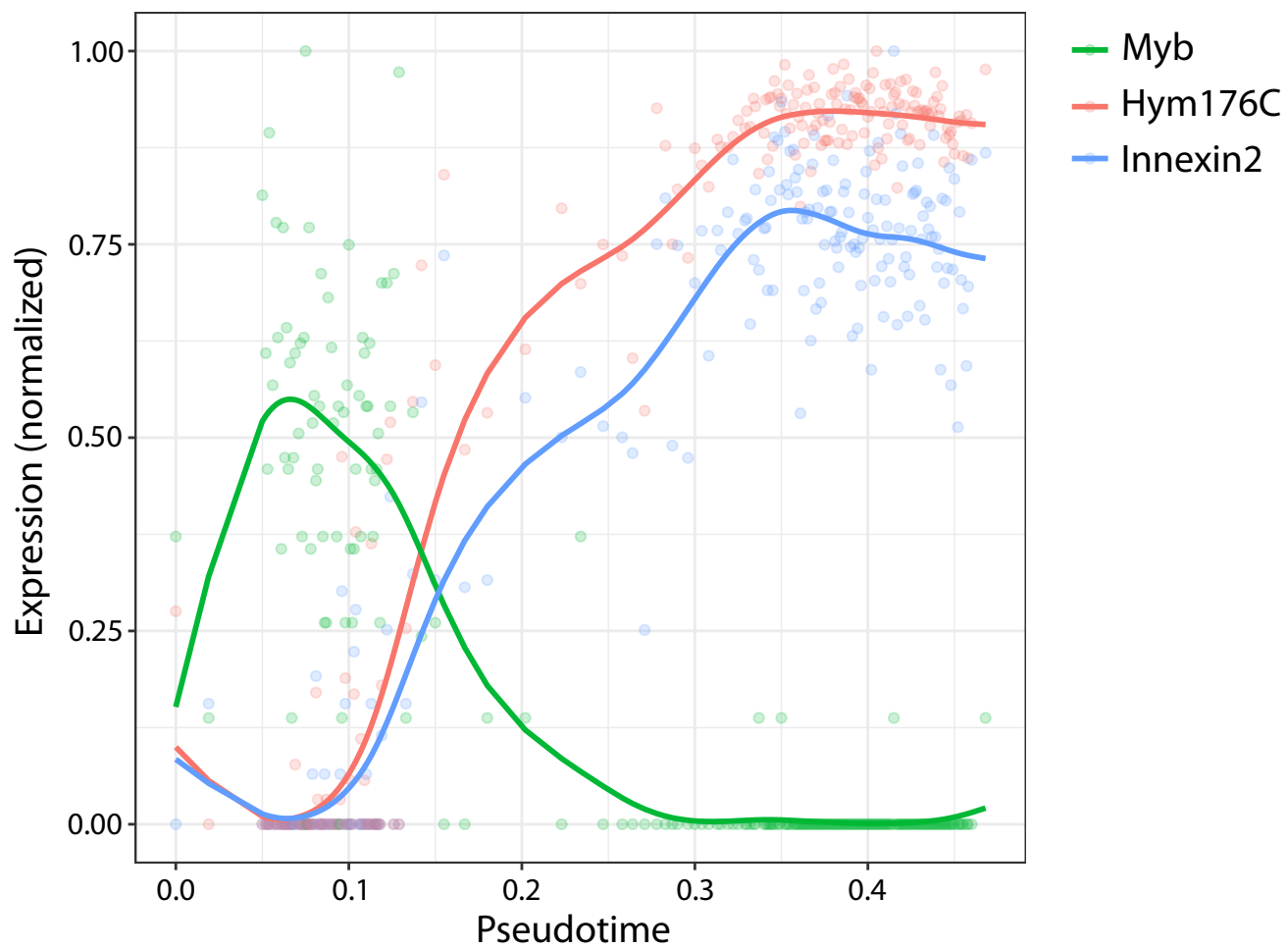

Supplement: Supplementary file 3 — Supplementary Material 3: Supplemental Figure 2. Dynamics of Innexin2, Myb, andHym176C in pseudotime for differentiation (public data from [21]). Left to right on the x axis corresponds to stages in differentiation. Y axis corresponds to log-normalized feature count scaled from 0 to 1 for comparison. [file 13064_2024_194_MOESM3_ESM.pdf]

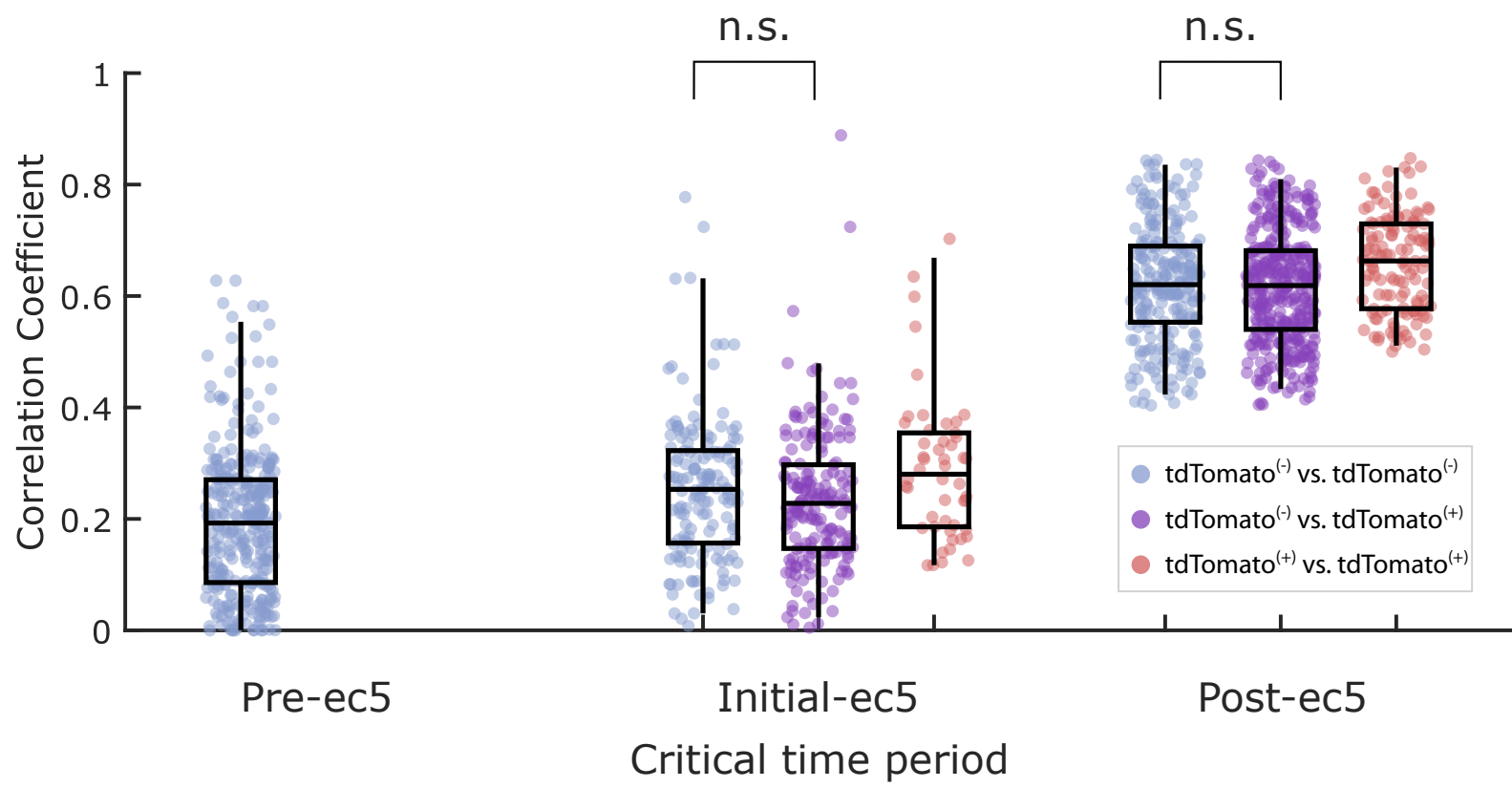

Supplement: Supplementary file 4 — Supplementary Material 4: Supplemental Figure 3. Correlation coefficient comparison between time points and between cell groups. Note that in the pre-ec5 time point, there are only tdTomato-negative neurons. Top line of the box corresponds to the third quantile (75%), middle line of the box corresponds to the median (50%), and the bottom line of the box corresponds to the first quantile (25%). The higher end of the whisker corresponds to 98%, and the lower end of the whisker corresponds to 2%. Kruskal-Wallis test with post hoc Dunn-Sidak test was used to evaluate statistical significance. [file 13064_2024_194_MOESM4_ESM.pdf]
